# Machine Learning Approach for Early Lactation Mastitis Diagnosis Using Total and Differential Somatic Cell Counts

**DOI:** 10.3390/ani15081125

**Published:** 2025-04-13

**Authors:** Alfonso Zecconi, Francesca Zaghen, Gabriele Meroni, Flavio Sommariva, Silvio Ferrari, Valerio Sora

**Affiliations:** 1 Department of Biomedical, Surgical and Dental Sciences, School of Medicine, University of Milano, Via Pascal 36, 20133 Milan, Italy; francesca.zaghen@unimi.it (F.Z.); gabriele.meroni@unimi.it (G.M.); valerio.sora@unimi.it (V.S.); 2 Department of Clinical and Community Sciences, School of Medicine, University of Milan, Via Celoria 22, 20133 Milan, Italy; 3 Associazione Regionale Allevatori Lombardia, Via Kennedy 30, 26013 Crema, Italy; f.sommariva@aral.lom.it (F.S.); silviof58@gmail.com (S.F.)

**Keywords:** mastitis, antimicrobial resistance, early diagnosis, differential cell count, machine learning

## Abstract

Dairy herds around the world are undergoing several changes. Herd sizes are increasing, as are both milk yield and quality. The implementation of new technologies in various domains of dairy production is leading to an increase in the quantity of data available. This, in turn, creates a need to extract useful information from these data to improve production efficiency. This paper presents the findings of a preliminary study that utilizes a machine learning (ML) approach to assess the accuracy of somatic cell count (SCC) and neutrophils + lymphocytes count/mL (PLCC) in identifying cows at risk of developing intramammary infection (IMI) due to major pathogens. These pathogens (MajPs) include *S. aureus, S. agalactiae*, *S. uberis*, and *S. dysgalactiae*. This study identified these pathogens either by real-time PCR (qPCR) methods or by conventional bacteriology, following the cows’ calving process. This study encompassed a total of 424 cows and 1696 quarter milk samples. A comparison of the two methods revealed significant disparities in the prevalence of MajPs, with the qPCR method demonstrating a higher prevalence than conventional bacteriology. However, the prevalence of negative results was comparable, with both methods yielding approximately 71.0% and 72.1%, respectively. The comprehensive results of this study substantiated that all the cellular markers exhibited the most accurate when MajP IMI was diagnosed using quarter milk samples, but this result is mainly due to the very high specificity. The cellular markers exhibited nearly equivalent performance, irrespective of the ML algorithm employed. The findings indicate that approaches based on SCC or PLCC may be useful for identifying healthy cows or quarters. However, it is essential to confirm all “non-negative” results through subsequent analysis within 7–15 days to ensure accuracy. However, further studies are necessary to enhance diagnostic accuracy.

## Simple Summary

The development of new technologies in many areas, including milk production, has significantly increased the quantity of data available and the need to extract useful information from these data to improve production efficiency. This paper reports the results of applying machine learning to detect cows at risk of intramammary infections caused by major pathogens based on total and differential somatic cell counts. The results confirm that these markers are useful in identifying infected cows and that the machine learning approach efficiently compares the accuracy of the diagnosis.

## 1. Introduction

Dairy herds worldwide are currently experiencing a series of changes that present both challenges and opportunities. Herd sizes are increasing, as are milk yield and quality [1]. New technologies have been implemented in several areas of dairy production (e.g., robot milking, precise livestock farming) [2,3,4]. Concurrently, new regulations have been implemented related to the control of antimicrobial resistance and the improvement of welfare [5].

The proliferation of novel technologies across diverse domains, including milk production, has led to a substantial augmentation in the availability of data. This, in turn, increased the need to extract useful information from these to improve production efficiency. In the context of dairy production, insights derived from data analysis have the potential to enhance the efficacy of early diagnosis, particularly for mastitis, thereby optimizing treatment protocols and reducing antimicrobial usage, ultimately enhancing cow welfare.

This analytical process was described as “knowledge discovery”, defined as the “non-trivial extraction of implicit, previously unknown and potentially useful information from data” [6]. One of the major components of the knowledge discovery process is data mining, which may be defined as “finding the existing patterns in data, which is the base for the further analyses and statistical processes, including machine learning (ML)” [7]. These techniques can analyze large quantities of data to predict different outcomes, including diseases, and dairy production has been applied to ketosis, lameness, and heat stress [8,9]. Recently, an ML approach was also applied to mastitis diagnosis in cows, buffaloes, and sheep [10,11,12,13,14,15,16].

The availability of differential somatic cell count (DSCC) on milk samples may be considered an additional source of information that could augment our capacity to diagnose subclinical mastitis [16,17]. Furthermore, DSCC data may be incorporated into the machine learning (ML) approach. This latter approach, at the best of our knowledge, was never applied using DSCC and aiming to perform early mastitis diagnosis.

Within a project (FEASR 16.1 project MOOH) encompassing the development and application of contagious mastitis pathogens control and mastitis early diagnosis as a tool to reduce and rationalize antimicrobial usage in dairy herds, this paper presents the results of a preliminary study on the application of machine learning in detecting cows at risk of intramammary infections (IMIs) caused by the major pathogens (MajPs) *S. aureus*, *S. agalactiae*, *S. uberis*, and *S. dysgalactiae* after calving.

## 2. Materials and Methods

### 2.1. Herd and Cow Selection

This study considered 424 clinically healthy cows that calved from June 2023 to December 2023 from 12 dairy herds in the Lombardy Region and enrolled in the Italian Breeder Association (AIA) monthly individual milk test (DHI). The herd size ranged from 90 to 500 lactating cows and 95% of the cows were Italian Friesian. All the herds were on cubicles indoor, and cows were milked in a milking parlor.

### 2.2. Sample Collection

Individual cow sampling (MTR) was performed by certified methods currently applied by AIA at the laboratories of the Regional Breeders Association of Lombardy (ARAL) using Lactocorder™ (WMB AG, Balgach, Switzerland), delivered refrigerated to ARAL labs the same day, and analyzed within 30 h from sampling.

At the lab, an aliquot of 1 mL was taken from each sample and stored in a sterile tube to be analyzed to identify major pathogens by qPCR as described below:

Quarter milk samples (QMSs) were taken within 1–3 days after previous individual sampling following the procedure described by N.M.C., 2017 [17], and delivered refrigerated to ARAL laboratories.

### 2.3. Cellular Marker Analyses

Milk analyses on MTR samples included somatic cell count (SCC) and DSCC and were carried out on Fossomatic™ 7DC (Foss A/S, Hillerød, Denmark). The DSCC was assessed by the method described by Damm et al. [18]. This method allows for identifying the macrophages (MACs) and the combination of polymorphonuclear leukocytes (PMNs) and lymphocytes (LYMs) within a milk sample. DSCC is expressed as the combined proportion (%) of PMN and LYM in the overall count of milk cells.

### 2.4. Conventional Microbiological Analysis

Bacteriological analyses were performed by streaking 10 µL of QMS on Tryptic Soy Agar + 5% *v*/*v* defibrinated sheep blood, according to N.M.C., 2017 [17].

After incubation (18–24 h at 37 °C) the colonies recovered were identified by Vitek™ system (Biomerieux, Lion, France).

Based on the results of the bacteriological analysis, a quarter was classified similarly to a previous paper [19]. Briefly, a quarter sample was classified as positive for an IMI due to major pathogens when 1 or more colonies of *S. agalactiae* or *S. aureus* were isolated, or when 5 or more colonies of *S. uberis* and *S. dysgalactiae* were isolated. It was considered positive for an IMI due to other bacteria when 5 or more colonies of the same species of Gram-negative pathogens (*E. coli*, *Klebsiella* spp., other coliforms) were isolated, or when 10 or more colonies of the same genus (coagulase negative *Staphylococcus* species, other environmental *Streptococcus* species, *Enterococcus* species) were isolated. The cases positive for bacteria other than major pathogens were classified as other bacteria in the statistical analysis.

### 2.5. Real-Time PCR Analysis

A commercial diagnostic kit was used (Mastitis 4A kit; DNA Diagnostic A/S, Risskov, Denmark) following the producer’s instruction. This kit allows for bacterial DNA extraction, identification, and quantification of *S. aureus*, *S. agalactiae*, *S. uberis*, and *S. dysgalactiae* using qPCR. The reaction conditions of qPCR were as follows: 95 °C for 1 min, 40 amplification cycles at 95 °C for 5 s and 60 °C for 25 s. Cycle threshold (Ct) values were considered positive when the value were ≤37, as suggested by the manufacturer. The qPCR reactions were performed on a Stratagene Mx3005P (Agilent Technologies Inc., Santa Clara, CA, USA).

### 2.6. Statistical Analysis

Data were collected in a database including herdID, cowID, days in milk (DIM), results of the bacteriological analysis by quarter, SCC and DSCC, and a variable PLCC calculated by multiplying SCC × DSCC. This variable PLCC represents the total number of PMN + LYM/mL, and it may be considered a more accurate assessment of inflammatory response [20].

The model supplied to the ML software algorithms considered the following response variables, MajP identified by qPCR, MajP identified by conventional bacteriology, or all positive samples identified by conventional bacteriology, while explanatory variables were SCC, DSCC, or PLCC; DIM (three classes: A 5–15 DIM, B 6–45 DIM, and C 45–90 DIM); and parturition (2 classes: primiparous and pluriparous cows).

The machine learning approach was based on Orange software 3.38.1 [21]. This software is a Python-based tool for data mining and a machine learning suite, and it includes a set of widgets for data preprocessing, with features such as compute modeling, model comparison, and exploration methods [21,22,23].

**Figure 1:**
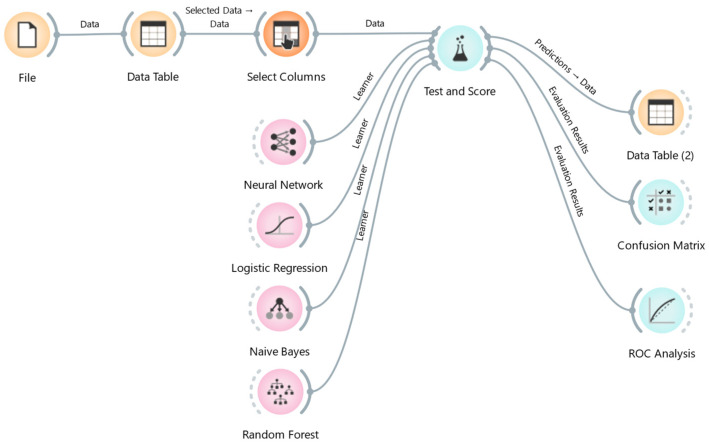
Diagrams of the workflow of Orange software applied to the milk sample data.

Orange Data Mining provides a vast range of data mining algorithms (DMAs). Naïve Bayes, decision tree, random forest, and logistic regression were used in this study [23,24] after a preliminary analysis led to the exclusion of algorithms with poorer performance. Figure 1 describes the workflow of the ML process. The analysis was performed applying a “leave-one-out” method, which randomly splits the data into the training and testing, holding out one instance at a time, inducing the model from all others and then classifying the instances. This method was selected because it is very stable and reliable.

### 2.7. Diagnostic Parameters

For each DMA, the following parameters were calculated:

- Area under the curve (AUC) of the ROC curve: it represents the degree or measure of separability; the higher the AUC, the better the model is at predicting the true status of the sample (positive/negative).
- Accuracy: expressed as a proportion of correctly classified subjects [true positive (TP) + true negative (TN)] among all subjects.
- Sensitivity (Se): the proportion of TP/[TP + false positive (FP)].
- Specificity (Sp): the proportion of TN/[false negative (FN) + TP].
- Positive predictive value (PPV): TP/(TP + FN).
- Negative predictive value (NPV): TN/(TN + FP).

## 3. Results

### 3.1. Data Description

The dataset containing all the information on cows, cell counts, and microbiological analysis (conventional and qPCR) was checked to identify missing data, such as the absence of SCC or DSCC, and for bacteriologically contaminated samples. All the records with these latter features were discarded and the final database included 424 valid records for cows and 1696 for quarter milk samples. The cellular marker data are summarized in Table 1.

The SCC, DSCC, and PLCC mean values were very close among the three DIM periods, and the lower values were observed in period B. Primiparous cows, as expected, showed lower means for cellular markers when compared to pluriparous ones, even if the values between these two groups were relatively close.

Table 2 reports the distribution of qPCR-positive results for *S. agalactiae*, *S. aureus*, *S. uberis*, and *S. dysgalactiae* (MaJPs), classified in the three lactational periods. The frequency of negative samples was higher in period B, supporting the lower cellular mean values observed, and in the same period, we had the lowest frequency for *S. agalactiae*, *S. uberis*, and *S. dysgalactiae*, whereas *S. aureus* frequency showed a marked increase as DIM increased. The *S. uberis* frequency was higher than the other pathogens, particularly in the period up to 45 DIM.

**Figure 2:**
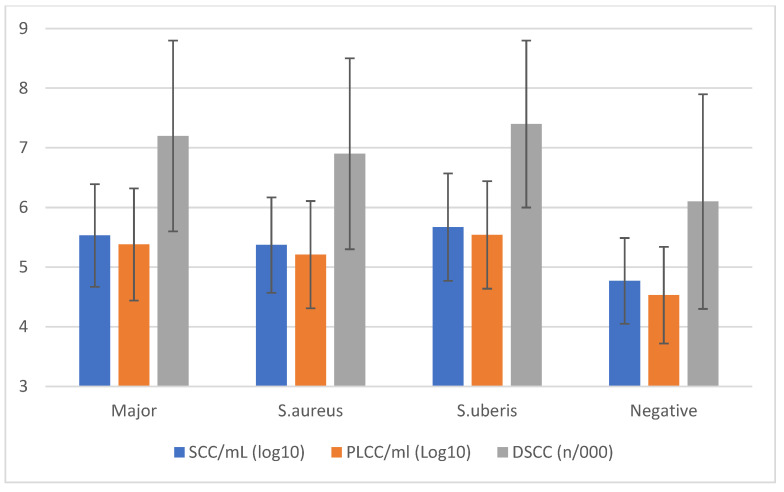
Cellular markers’ means (±std. dev.) classified by the results of qPCR analysis on individual milk samples.

The comparison of cellular markers (Figure 2) based on the udder health status confirmed that MajP increased the mean values by nearly 1 log compared to negative samples.

When data were classified by the number of parturitions (Table 3), the results showed that nearly 80% of samples from primiparous cows were negative, while only 67% of pluriparous cows were free from MajP infections. In this latter group, *S. uberis* and *S. aureus* IMI frequencies nearly doubled compared to primiparous cows. The few *S. agalactiae* IMIs observed were all related to pluriparous cows.

When quarter milk samples were considered (Table 4), the results showed nearly identical overall frequencies of negative samples compared to qPCR results, even if there were differences in the different DIM classes. However, the frequency of MajPs was lower than in individual samples.

*S. aureus* was overall the most frequent pathogen recovered among the MajPs with a prevalence of 2.6%, but this value was mainly due to the frequency observed in period C (4.2%). At the same time, *S. uberis* showed an overall prevalence of 2.0% and frequency of 1.7%, 2.2%, and 2.1%, respectively, in period A, B, and C. These two pathogens represented >90% of the MajPs isolated.

**Figure 3:**
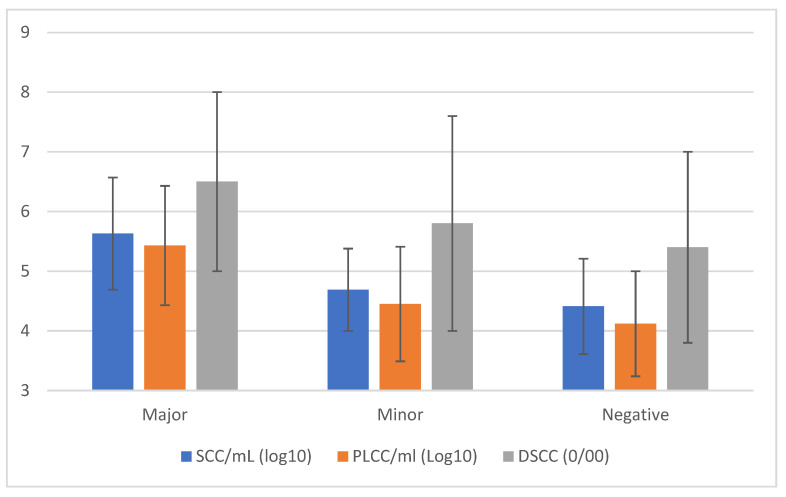
Cellular markers’ means (±std. dev.) classified by the results of quarter milk samples analysis by conventional microbiological methods.

The results of SCC, DSCC, and PLCC (Figure 3) based on the outcome of the microbiological analysis confirm that the presence of MajP is associated with an inflammatory status with values higher than in quarter positives for minor pathogens or bacteriological negatives. The mean values for the three different cellular markers were very similar in these latter two cases.

When data were classified by the number of parturitions (Table 5), the frequency of IMI in primiparous cows was lower than in pluriparous cows, but the difference between the two groups was smaller than those observed for individual milk samples. It is worth noting that the increase in overall IMI frequency was mainly due to the increase in MajP frequency.

### 3.2. Machine Learning Analysis

The data were analyzed by data-mining algorithms, considering as response variables the positive results at qPCR analysis of individual milk samples, the positive results for MajPs at the conventional bacteriological analysis of QMS or the positive results at the conventional bacteriological analysis of QMS.

Table 6 reports the results of the qPCR analysis, and an accuracy in the range 0.690–0.774 was observed among the different algorithms and cellular markers. Logistic regression showed the highest values among algorithms and SCC and PLCC among the cellular markers. Overall sensitivity was small, with values of 57.6% only for PLCC and DSCC when logistic regression was applied. The specificity was higher with values of about 79% for logistic regression and SCC and PLCC, while the highest values were observed for random forest with values around 81%. Subsequently, NPV was >95%, while PPV was <20% for most algorithms and cellular markers.

When conventional bacteriological analysis on quarter milk samples was considered (Table 7) in identifying MajP, the results differed from the analogous analysis based on qPCR. Indeed, the accuracy was always close to 95%, independently from the algorithm and the cellular markers. However, this result is only due to a very high specificity, while sensibility was very poor.

When a positive bacteriological result was considered instead of only MajP (Table 8), the results showed an overall accuracy in the range 0.657–0.733, with the higher values observed for logistic regression and neural network algorithms and PLCC and SCC.

Logistic regression on PLCC and SCC as cellular markers showed the highest sensitivity values, respectively, 61.4% and 64.3%, while specificity was around 74% for both markers. These values led to a low PPV, but an NPV > 95% for both PLCC and SCC.

Finally, DSCC showed the poorest performance for all four algorithms and the three outcomes considered.

## 4. Discussion

### 4.1. Intramammary Infections

The early assessment of udder health after calving has become increasingly important as selective dry-cow therapy is widely applied in many countries. Indeed, it facilitates the identification of infected animals, thereby enabling the judicious and rational administration of antimicrobial therapy, which in turn reduces the risk of antimicrobial resistance (AMR) [25]. This approach is relatively labor-intensive (milk sampling) and may be expensive (microbiological analysis). Additionally, its implementation in dairy herds with >200 cows can pose significant challenges.

The availability of new technologies such as DSCC and qPCR on the one hand, and new approaches to data analysis such as ML on the other hand, paved the way for the development of new protocols to identify cows at risk. A previous study supported this approach by showing that early diagnosis based on DSCC is cost-effective [19].

The present study aimed to improve this approach by evaluating whether qPCR and ML analytical approaches can increase the accuracy and efficiency of early mastitis diagnosis.

The analysis of the results of mastitis diagnosis on MTR samples and QMS allowed us to identify some critical points. Indeed, the analysis of the cellular and microbiological data showed that the different time windows influenced the result. The mean values observed for both SCC and PLCC markers in the first two periods considered (5–15, 16–45 DIM) were significantly different for the third period (46–90 DIM). In addition, the values in period B (16–45 DIM) were the lowest. Similarly, IMI prevalence was lowest in the first two DIM intervals for both MTR and QMS. These differences are very likely due to the increase of teat exposure to bacteria as the number of milking increases.

The results of the comparison between MTR samples (qPCR) and QMS (conventional bacteriology) showed, as expected, large differences in the prevalence of MajPs as a whole and when considering the individual species. In fact, the prevalence of MajPs in MTR was 16.1% compared to 4.6% in QMS. Despite these differences, the prevalence of negative MTR or QMS was very close at 71.0% and 72.1%, respectively. The differences in MajPs may be explained, at least in part, by the different diagnostic methods, and it can be argued that qPCR identifies DNA and not live bacteria, and that some of the positive results may be due to environmental contamination (in the case of *S. uberis* and *S. dysgalactiae*). However, this argument does not apply to infectious bacteria. The qPCR was advantageous to identify the presence of IMI when the bacterial concentrations were below the detection limits of conventional bacteriology or the samples were contaminated [26,27]. Therefore, the analysis of the association of cellular markers with infection status may be useful to identify the presence of an evolving infection.

### 4.2. Machine Learning Approach

The availability of automated instruments to measure DSCC in milk samples has greatly improved our ability to diagnose mastitis [19,28,29,30]. When applied to MTR samples, this analysis generates a large quantity of data that may be analyzed using the ML approach. In fact, ML has been used to diagnose clinical and subclinical mastitis [10,13,31], but DSCC was not included in these studies.

The different algorithms applied to the same set of data showed, as expected, some differences. Indeed, overall logistic regression and neural network performed better than naïve Bayes and random forest. Different outcomes related to the algorithm applied were expected and already observed [13], and they may also be explained by the characteristics of the database considered. The capability of Orange tool and other ML software to compare different algorithms is pivotal to identify the best algorithm and model to be applied on a routine basis, when the ML approach is used to diagnose diseases.

Our study included a relatively small number of data, but the sample size is comparable or greater than other studies on the diagnostic of dairy cows [8,32] or human diseases [33,34].

The overall results confirmed that all cellular markers showed the highest accuracy values when MajP IMI was diagnosed using quarter milk samples. In fact, regardless of the algorithm used, the accuracy was always >90%. This result was due to the very high specificity observed (>95%), while the sensitivity was very low and absent in most cases. This result was expected due to the low frequency of MajP observed in QMS. Moreover, the comparison with qPCR analysis suggests that conventional bacteriological analysis may fail to find many infections in the early lactating period. When MTR and qPCR were considered, the accuracy ranged from 63–74%, depending on the algorithm, with sensitivity in the range of 30–57% and specificity in the range of 78–82%. In both cases, MTR and QMS, the NPV was very high, reaching 100% in some cases. The observed sensitivity and specificity values were similar to the results of other studies [10,14], but in contrast to those observed by Ebrahimi et al. [35].

DSCC had the lowest performance among the cellular markers, and this is not surprising, since it gave information on the proportions of cell, but not on their number. Indeed, only the combined use of both SCC and DSCC showed to be worthwhile [36,37], whereas SCC and PLCC had nearly equal performance, regardless of the algorithm used. This result has useful implications in practice. Indeed, DSCC and PLCC are not available without the specific counting instrument (Fossomatic™ 7DC, Foss A/S, Hillerød, Denmark). However, SCCs are always available when a DHI program is applied, as is the case in most countries with large milk production. Therefore, in the latter case, the identification of cows at risk for MajP IMI is possible, while when PLCC can be calculated, it is additional information to be applied at cow level to better define the cow’s udder health status [20]. The results of this study confirm that the frequency of IMI due to MajPs had the highest level when detected in the period 45–90 DIM, suggesting the importance of an early diagnosis to prevent the insurgence of clinical mastitis. Finally, these results suggest that approaches based on SCC or PLCC evaluated with ML may help to identify healthy cows or quarters, while all “non-negative” cows should be confirmed by a subsequent analysis in the following 7–15 days.

## 5. Conclusions

Early and accurate diagnosis of disease is important in any field of medicine. In the case of dairy cow IMI, such diagnosis has several positive implications: higher cure rate, rational and reduced use of antimicrobials, improved cow welfare, and higher milk yield and quality. Early diagnosis is particularly important after calving, when cows are exposed to pathogens due to their compromised immune status as a result of calving. Changes in dairy herd management include new approaches and technologies that could help identify cows at risk for MajP IMI. The results of this pilot study under field conditions confirm that cellular markers related to total and differential cell counts may be useful in identifying these cows and that the ML approach is useful in comparing the accuracy of the different diagnostic models. However, further studies are needed to increase the diagnostic accuracy.

## Figures and Tables

**Table 1:** Description of sample characteristics: cellular markers mean values ± standard deviation of cow classified by days in milk at sampling and by number of parturitions.

	N	SCC ^1^ ± Std. Dev (Log_10_/mL)	DSCC ^2^ ± Std. Dev (%)	PLCC ^3^ ± Std. Dev (Log_10_/mL)
**Lactation period**				
A (5–15 d)	88	4.98 ± 0.22 ^a,4^	63.1 ± 17.7 ^a^	4.76 ± 0.90 ^a^
B (16–45 d)	111	4.78 ± 0.83 ^a^	61.9 ± 18.1 ^a^	4.55 ± 0.93 ^a^
C (46–90 d)	225	5.00 ± 0.82 ^b^	64.4 ± 18.3 ^a^	4.79 ± 0.92 ^b^
**Parturition**				
Primiparous	133	4.81 ± 0.66 ^a^	63.8 ± 19.5 ^a^	4.60 ± 0.73 ^a^
Pluriparous	291	5.01 ± 0.92 ^b^	64.0 ± 15.2 ^a^	4.80 ± 1.02 ^b^

^1^ SCC = somatic cell count. ^2^ DSCC = differential cell count. ^3^ PLCC = SCC × DSCC. ^4^ columns with different superscripts (letters) are statistically different (α = 0.05).

**Table 2:** Udder health status: distribution of intramammary infections based on qPCR analysis targeting *S. agalactiae*, *S. aureus*, *S. uberis*, and *S. dysgalactiae*, classified by days in milk.

Lactation Period	*S. aureus*	*S. agalactiae*	*S. uberis*	*S. dysgalactiae*	Negative
A (5–15 d)	2.3	2.3	17.0	4.5	73.9
B (16–45 d)	5.4	0.0	8.9	0.9	84.8
C (46–90 d)	14.5	0.0	19.3	3.1	63.1
Total	9.6	0.5	16.1	2.8	71.0

**Table 3:** Udder health status: distribution of intramammary infections based on qPCR analysis targeting *S. agalactiae*, *S. aureus*, *S. uberis*, and *S. dysgalactiae*, classified by number of parturitions.

Parturition	*S. aureus*	*S. agalactiae*	*S. uberis*	*S. dysgalactiae*	Negative
Primiparous	6.8	0.0	10.5	3.0	79.7
Pluriparous	11.0	0.7	18.6	2.7	67.0
Total	9.6	0.5	16.1	2.8	71.0

**Table 4:** Udder health status: distribution of positive samples from conventional microbiological analysis of quarter milk samples, classified by days in milk.

Lactation Period	Quarter (N)	MajP ^1^	Other Pathogens	Negative
A (5–15 d)	352	1.9%	18.8%	79.3%
B (16–45 d)	444	2.9%	22.3%	74.8%
C (46–90 d)	900	6.5%	25.6%	67.9%
Total	1696	4.6%	23.3%	72.1%

^1^ MajP: major pathogens S. agalactiae, S. uberis, S. dysgalactiae, and S. aureus.

**Table 5:** Udder health status: distribution of positive samples from conventional microbiological analysis of quarter milk samples, classified by number of parturitions.

Parturition	Quarter (N)	MajP ^1^	Other Pathogens	Negative
Primiparous	648	2.6%	22.8%	74.6%
Pluriparous	1048	5.9%	23.6%	70.5%
Total	1696	4.6%	23.3%	72.1%

^1^ MajP: major pathogens S. agalactiae, S. uberis, S. dysgalactiae, and S. aureus.

**Table 6:** Results of the four algorithms’ analysis in identifying major pathogens by qPCR in individual milk samples.

Model	Parameter	AUC ^4^	Accuracy	Sensitivity	PPV ^5^	Specificity	NPV ^6^
Logistic regression	PLCC ^1^	0.740	0.774	57.6%	17.6%	78.9%	96.0%
	SCC ^2^	0.743	0.774	57.6%	17.6%	78.9%	96.0%
	DSCC ^3^	0.665	0.763	0.0%	0.0%	100%	76.3%
Neural network	PLCC	0.733	0.760	42.3%	20.4%	78.7%	91.4%
	SCC	0.739	0.765	51.2%	19.4%	79.0%	93.7%
	DSCC	0.651	0.760	33.3%	0.9%	76.3%	99.4%
Naïve Bayes	PLCC	0.711	0.758	48.1%	24.1%	79.6%	91.9%
	SCC	0.717	0.758	48.1%	24.1%	79.6%	91.9%
	DSCC	0.662	0.763	0.0%	0.0%	76.3%	100.0%
Random forest	PLCC	0.684	0.745	45.6%	38.0%	81.6%	85.9%
	SCC	0.656	0.727	40.9%	33.3%	80.4%	85.0%
	DSCC	0.630	0.690	30.6%	24.1%	77.8%	83.0%

^1^ PLCC = SCC × DSCC/mL; ^2^ SCC = somatic cell count/mL; ^3^ DSCC = differential cell count/mL; ^4^ AUC = area under the curve of the ROC curve; ^5^ PPV = positive predictive value; ^6^ NPV = negative predictive value.

**Table 7:** Results of the four algorithms’ analysis in identifying major pathogens by conventional microbiology on quarter milk samples.

Model	Parameter	AUC ^4^	Accuracy	Sensitivity	PPV ^5^	Specificity	NPV ^6^
Logistic regression	PLCC ^1^	0.816	0.952	0.0%	0.0%	95.3%	99.9%
	SCC ^2^	0.821	0.952	0.0%	0.0%	95.3%	99.8%
	DSCC ^3^	0.686	0.953	n.a. ^7^	0.0%	95.3%	100.0%
Neural network	PLCC	0.806	0.952	0.0%	0.0%	95.3%	99.9%
	SCC	0.811	0.951	16.7%	1.2%	95.4%	99.7%
	DSCC	0.658	0.953	n.a.	0.0%	95.3%	100.0%
Naïve Bayes	PLCC	0.770	0.953	n.a.	0.0%	95.3%	100.0%
	SCC	0.780	0.953	n.a.	0.0%	95.3%	100.0%
	DSCC	0.639	0.953	n.a.	0.0%	95.3%	100.0%
Random forest	PLCC	0.723	0.933	21.5%	16.5%	96.0%	97.1%
	SCC	0.711	0.943	32.7%	20.0%	96.2%	98.0%
	DSCC	0.602	0.951	0.0%	0.0%	95.3%	99.7%

^1^ PLCC = SCC × DSCC/mL; ^2^ SCC = somatic cell count/mL; ^3^ DSCC = differential cell count/mL; ^4^ AUC = area under the curve of the ROC curve; ^5^ PPV = positive predictive value; ^6^ NPV = negative predictive value. ^7^ n.a. = not available.

**Table 8:** Results of the four algorithms’ analysis in identifying all pathogens by conventional microbiology on quarter milk samples.

Model	Parameter	AUC ^4^	Accuracy	Sensitivity	PPV ^5^	Specificity	NPV ^6^
Logistic regression	PLCC ^1^	0.638	0.728	61.4%	16.9%	73.8%	95.7%
	SCC ^2^	0.637	0.732	64.3%	17.0%	73.9%	96.1%
	DSCC ^3^	0.595	0.710	n.a. ^7^	0.0%	71.0%	100.0%
Neural network	PLCC	0.657	0.733	60.5%	22.9%	74.9%	93.9%
	SCC	0.653	0.733	60.7%	22.5%	74.8%	94.0%
	DSCC	0.621	0.716	55.8%	10.0%	72.5%	96.7%
Naïve Bayes	PLCC	0.618	0.713	51.6%	18.6%	73.6%	92.9%
	SCC	0.616	0.710	n.a.	0.0%	71.0%	100.0%
	DSCC	0.587	0.710	n.a.	0.0%	71.0%	100.0%
Random forest	PLCC	0.586	0.657	39.6%	34.8%	74.6%	78.3%
	SCC	0.595	0.683	43.1%	29.2%	74.4%	84.3%
	DSCC	0.543	0.660	35.1%	20.1%	72.2%	84.8%

^1^ PLCC = SCC × DSCC/mL; ^2^ SCC = somatic cell count/mL; ^3^ DSCC = differential cell count/mL; ^4^ AUC = area under the curve of the ROC curve; ^5^ PPV = positive predictive value; ^6^ NPV = negative predictive value. ^7^ n.a. = not available.

## Data Availability

The data are not publicly available due to privacy.
